# Case of Oral Surgery

**Published:** 1879-03

**Authors:** H. M. Grant


### ARTICLE V.

### *Case of Oral Surgery.*

BY H. M. GRANT, M. D., D. D. S.

Reported to the Abingdon Academy of Medicine, February 3rd, 1879.

About the first of November, 1878, I was consulted by Mr. J. H. D., aged 23, who was suffering from an immense tumor, situated in the roof of his mouth and almost filling the oral cavity. The history of the case as given me by the patient and his family was as follows:

In September, 1873, he received a blow upon the right superior maxillary, by a fall, by which the second superior molar tooth of that side was broken off, and the palatine fang driven up into the roof of the mouth, within the body of the bone. Some eight months afterwards, he felt a hard tumor or protuberance near the mesial line of the hard palate, which continued to increase in size up to the time I first saw him.

Upon examination of the case, I found the tumor to be tubelated or of irregular form, very firm in texture, filling the entire oral cavity, its base extending from the palatine surface of the incisor and cuspid teeth (which were all displaced, being pressed forward, until their position was almost horizontal with the plane of the corronal points of the upper teeth in their natural position,) to the posterior border of the hard palate, and base of the azygos uvulæ

muscles, and laterally from the palatine border of the alveolar of the bicuspid, and molar teeth on the right side to the same point on the left—encroaching upon the molar teeth and bicuspids, until they were also forced out of position, and some of them protuding through the cheek on either side. The jaws being distended to such an extent from the growth and size of the tumor, that his countenance was most hideously disfigured, and the patient an object of commiseration.

The integuments and mucous membrane had assumed a livid and conjested appearance; but no ulcerative action had taken place, except from the laceration of the cheek by the protruding teeth on either side.

Having determined upon excision, the following mode of procedure was adopted. The patient was given vi. grs. quinia one hour before the operation was performed to prevent shock. He was then brought under the influence of chloride ether, consisting of 2 oz. of the former to one of the latter. The upper teeth were then all extracted, the first and second molars of the left side having been extracted some years before.

An incision was then made from the alveolar border of the central incisors backward along the mesial line, to the posterior border of the hard palate or periphery of the base of the tumor; another incision was made from a point opposite the anterior approximal surface of the first molar on the right side, to the same point on the left, carried down through the mucous membrane and integument, as deep as the texture of the tumor would admit. The flaps, so to speak, were then dissected up and carried to, and held to either side by small flexible tenaculum prepared for the purpose, and attached to adhesive strips on the outside of the oral cavity. The body of the tumor was thus brought to view. Then with a dental burring engine (Morrison's,) with a small steel circular saw or disk, the substance of the tumor was then cut away in sections down to the hard palate or bone, being very careful to preserve its surface intact and uninjured. After the tumor was removed, the surface of the bone was thoroughly scraped with round pointed scrapers with cutting edges. The four flaps of mucous membrane trimmed to fit, being careful to shape them to correspond with the arched roof of the mouth and brought together and held in juxtaposition by interrupted sutures made with silver wire, three on either side of the central point of union of the flaps, and held in position in contact with the arch, by means of a spiral roll of lint. Kept in position by means of a small spring, made of gold plate, so bent or curved to fit the arch, and attached to a support resting upon the crowns of the second lower molar teeth. The jaws brought into their natural position and bound by a bandage, such as is used in fracture of the lower jaw. A piece of semi—vulcanized rubber, being interposed as a support for the lower jaw, and for the admission of nourishment during the healing process.

The hemorrhage was but slight and controlled by Monsel's persulphate iron. The patient soon recuperated from the effects of the anæsthetic and vi. gr. of quinia was again administered. The oral cavity thoroughly washed with a solution of carbolic acid alternated with phenole sodique for 10 days. The wound healed rapidly and kindly and I am happy to state my patient has entirely recovered, and will be supplied with an upper denture, as soon as the absorption of the alveolar process has taken place.

I think that it was a fibro-cellular tumor; its weight two hours after excision was 4 oz. and 8 gr. Its texture was very firm, so much so as to resist the action of the knife, and could only be removed with a small circular saw or disk, and preserve the arch or roof of the mouth intact.

